# Functional traits explain crayfish invasive success in the Netherlands

**DOI:** 10.1038/s41598-021-82302-4

**Published:** 2021-02-02

**Authors:** Tiedo van Kuijk, Jacobus C. Biesmeijer, Berry B. van der Hoorn, Piet F. M. Verdonschot

**Affiliations:** 1 Naturalis Biodiversity Center, P.O. Box 9517, 2300 RA Leiden, The Netherlands; 2 Institute for Biodiversity and Ecosystem Dynamics, University of Amsterdam, Amsterdam, The Netherlands; 3 Institute of Environmental Sciences, Leiden University, Leiden, The Netherlands; 4 Wageningen Environmental Research, P.O. Box 47, 6700 AA Wageningen, The Netherlands

**Keywords:** Freshwater ecology, Invasive species

## Abstract

Biological invasions by nonindigenous species can have negative effects on economies and ecosystems. To limit this impact, current research on biological invasions uses functional traits to facilitate a mechanistic understanding of theoretical and applied questions. Here we aimed to assess the role of functional traits in the progression of crayfish species through different stages of invasion and determine the traits associated with invasive success. A dataset of thirteen functional traits of 15 species currently occurring or available for sale in the Netherlands was evaluated. Six of these crayfish appeared invasive. Important traits distinguishing successful from unsuccessful invaders were a temperate climate in the native range, a medium to high egg count and producing more than one egg clutch per year. The most successful invaders had different functional trait combinations: *Procambarus clarkii* has a higher reproductive output, can migrate over longer distances and possesses a higher aggression level; *Faxonius limosus* is adapted to a colder climate, can reproduce parthenogetically and has broader environmental tolerances. Using a suit of functional traits to analyse invasive potential can help risk management and prevention. For example, based on our data *Procambarus virginalis* is predicted to become the next successful invasive crayfish in the Netherlands.

## Introduction

Over the past decades worldwide trade and traffic have greatly increased, leading to the introduction of many species into ecosystems beyond their native range^1^. Nonindigenous species that established new populations outside their native range have been widely recognized to have potential negative impacts on local ecosystems and are generally regarded as one of the largest threats to biodiversity besides habitat destruction^2,3^. Their impacts include competition, grazing, predation and introduction and spread of diseases^4,5^. In addition to their potential contribution to species extinctions^6^, nonindigenous species may also have severe economic impacts: between 1992 and 2006 the European Union spent over 130 million euro on projects dealing with nonindigenous species^7^.

Aquatic ecosystems are considered more vulnerable to species introductions than terrestrial systems, due to active (e.g. fish stocking) and passive (e.g. releasing ballast water) import of nonindigenous species^8,9^. The United Nations Food and Agriculture Organization’s Database of Invasive Aquatic Species states that on average 63% of aquatic species become established after introduction^10^. One of the most successful taxa currently invading freshwater systems is decapods, with 46% of the nonindigenous species in Europe being invasive^11^. In the Netherlands, decapods represent 18% of about 66 recorded nonindigenous macroinvertebrate species^12^. Within the decapods, crayfish are well known for their invasive potential and are currently displacing native crayfish throughout Europe^13,14^. *Procambarus clarkii*, for instance, is considered a highly invasive species throughout Europe with negative impacts on invaded ecosystems^15,16^. Currently, at least ten nonindigenous crayfish species have established populations in Europe^17^.

One third of the 100 worst aquatic invasive species^18^ originates from aquarium releases^19^. Additionally, worldwide sale of pet crayfish has grown substantially in recent decades: about 130 of roughly 600 different crayfish species were reportedly sold as pets^20^. In the Netherlands, nine crayfish species are commonly for sale^21^. In crayfish, functional traits such as bright coloration, smaller size, or single parent reproduction (parthogenesis) seem to contribute to their popularity as pets^20^. Research by Chucholl & Wendler^22^ showed that 67% of high risk crayfish sold in Germany possess traits attractive for pet owners. They found such functional traits can be directly related to long-term presence of nonindigenous species in the aquarium trade and concluded that higher risk crayfish are selected for introduction due to popular traits (Table 1). Similarly, Zeng et al.^23^ found species with large clutch sizes used for non-commercial harvesting (exploitation for food, recreation, and by hobbyists for personal aquariums) and bait to have increased risk of introduction.

**Table 1:** Invasion framework by Blackburn et al*.*^24^ with functional traits mentioned in literature as relevant to the barriers of each stage.

Invasion stage	Barrier	Associated traits	References
Transport (A)	Geography	Size of native range	^33,38,62^
Introduction (B)	Captivity/cultivation	Preference or tolerance of lentic habitats	^22,23,32,33^
		Able to reproduce at warm-water aquarium conditions	^22^
		Absence of specialized germination or hatching requirements	^26^
		Bright body coloration	^20,22^
		Small body size (when not brightly colored)	^22^
Establishment (C)	Survival	Wide environmental and climatic tolerances	^26,33,38,62^
		Generalist diet	^25,33,38^
	Reproduction	Single parent reproduction	^20,25,26,62^
		High reproductive potential	^23,25,26,32,33,38,62^
		Short generation time	^25,26^
		High growth rate	^26^
Spread (D)	Dispersal	Good dispersal	^25,26,33^
Fully invasive (E)	Environmental/competition	Ability to escape or survive natural enemies	^26^
		Long lived (resist mortality)	^28^
		Large size	^23,32,38^
		High competitive ability	^23,25,26,32,33^

Blackburn et al.^24^ suggested a unified framework for biological invasions that combines aspects of both plant and animal ecology and focuses on human-mediated invasions. This framework describes the progressive stages of an invasion from transportation to fully invasive, including specific barriers for each stage (Table 1). A nonindigenous species only progresses through successive stages of invasion when it possesses barrier-specific functional traits^11^. For example, successful invasive species often have traits such as wide environmental and climatic tolerances, a generalist diet, high reproductive potential and good dispersal ability^25,26^. By identifying species with traits associated with high invasive potential, invasions with negative impacts can be predicted and prevented more effectively^27^.

Several efforts were made to assess the functional traits associated with successful aquatic invaders from different taxa, including fish^28^, gammarids^29^, plants^30^ and marine crabs^31^. For crayfish, Larson & Olden^32^ classified the invasive potential of 77 species in the United States using a functional trait-based analysis. They identified species with a high latent risk to become invasive outside their native range, taking into account that certain traits can be more beneficial in specific stages of invasion. Their results showed that successful invasive crayfish were grouped together based on the traits large size, high fecundity and lentic, terrestrial and generalist habitat preferences. Zeng et al.^23^ determined the invasion status of 614 crayfish species according to the framework of Blackburn et al.^24^ and assessed which functional traits were associated with species progression to the stages of establishment and spread. Their analysis included human-associated traits such as non-commercial harvesting and ornamental trade. They found clutch size to be predictive of the spread stage. An invasiveness screening tool for fish was also adapted into the Freshwater Invertebrate Invasiveness Scoring Kit (FI-ISK) to assess the invasion risk of nonindigenous crayfish species in Italy, based partly on trait related information^33^.

This paper aims to address the role that functional traits play in the transition of nonindigenous crayfish through the stages of establishment and spread to become fully invasive, and to determine which traits are associated with invasive success. To achieve this, the distribution, relation to their environment and the functional traits of nonindigenous crayfish species currently present in the Netherlands were assessed. The traits of successful and unsuccessful invasive species were compared to determine which specific traits allowed them to pass through the stages of invasion according to the Blackburn framework. Finally, the current invasion status of crayfish in the Netherlands was compared to predictions of invasion risk by other studies. Based on previous research^22,23,32^ we expected successful invasive species to possess different traits than unsuccessful species, with specific combinations of traits being required to pass all invasion stages leading to invasive success.

## Results

### Crayfish distribution

**Figure 1:**
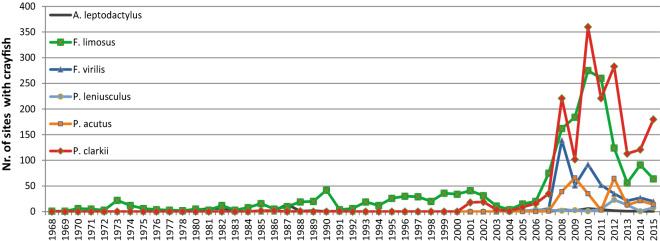
Number of sites with crayfish occurrences per year.

**Figure 2:**
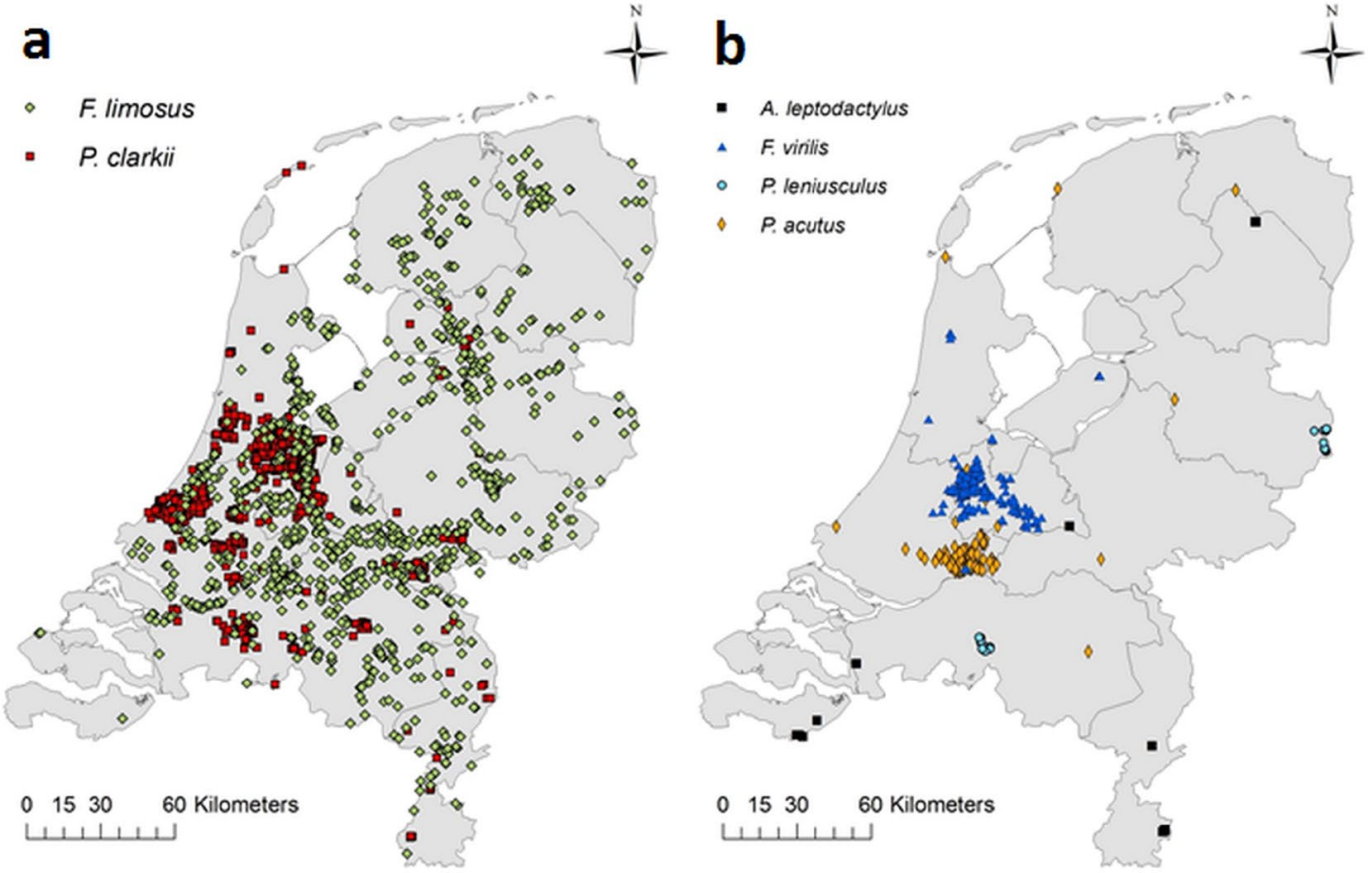
Distributions of (**a**) *F. limosus* and *P. clarkii* and (**b**) *A. leptodactylus*, *F. virilis*, *P. leniusculus* and *P. acutus* in the Netherlands. These maps were generated using ArcMap 10.2.2 (https://desktop.arcgis.com/en/arcmap/).

Several nonindigenous crayfish species were first observed in the Netherlands multiple decades ago, such as *Faxonius limosus, Astacus leptodactylus*, and *P. clarkii* (1968, 1978, and 1985, respectively) (Fig. 1). More recently introduced species were *Faxonius virilis* (2004), *Pacifastacus leniusculus* (2005), and *Procambarus acutus* (2007). The species found in the highest number of sites were *P. clarkii* and *F. limosus*, while *A. leptodactylus* and *P. leniusculus* were recorded in the lowest number of sites. *Pacifastacus leniusculus* was reported in two areas, each with multiple locations nearby. In contrast, *A. leptodactylus* had been found in multiple locations further apart from each other. *Faxonius virilis* and *P. acutus* were sighted at an intermediate number of sites. Although *F. virilis* and *P. acutus* hardly co-occurred, there was a similar pattern in sightings (Fig. 2b) as both occur mainly within a limited region with sparse sightings in other regions. Although *P. clarkii* and *F. limosus* both occurred at many sites, there is a clear difference between their spatial distributions (Fig. 2a). The former was found mainly in the mid-west of the Netherlands, while the latter was spread more evenly throughout the entire country. Finally, *P. acutus* showed relatively little overlap with *P. clarkii*, while *F. virilis* did occur partially over the same geographical area as *P. clarkii*.

### Occurrence in different water types

**Figure 3:**
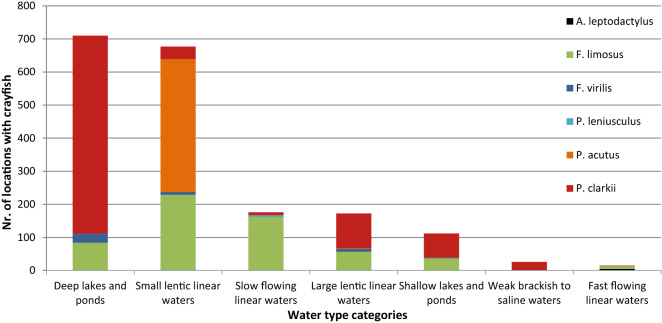
Number of sites per water type for each of the six crayfish species (data from 2007 to 2015).

The occurrence of crayfish in different water types is species specific (Fig. 3). *Procambarus clarkii* occurred in waterbodies of all types but was mostly present in both shallow and deeper lakes and ponds and in larger, linear, lentic waterbodies such as canals. In contrast, *P. acutus* was found only in small, linear, lentic waters (mainly man-made ditches and canals, all under 3 m deep and 15 m wide). While *F. virilis* predominantly occurred in deep lakes and ponds, *F. limosus* was distributed over multiple locations across all water types, except brackish and saline waters. Few crayfish were found in brackish and saline waters and fast flowing waters, only *A. leptodactylus* was found exclusively in these water types. *Pacifastacus leniusculus* occurred only in slow flowing waters.

### Crayfish clustered by traits

**Figure 4:**
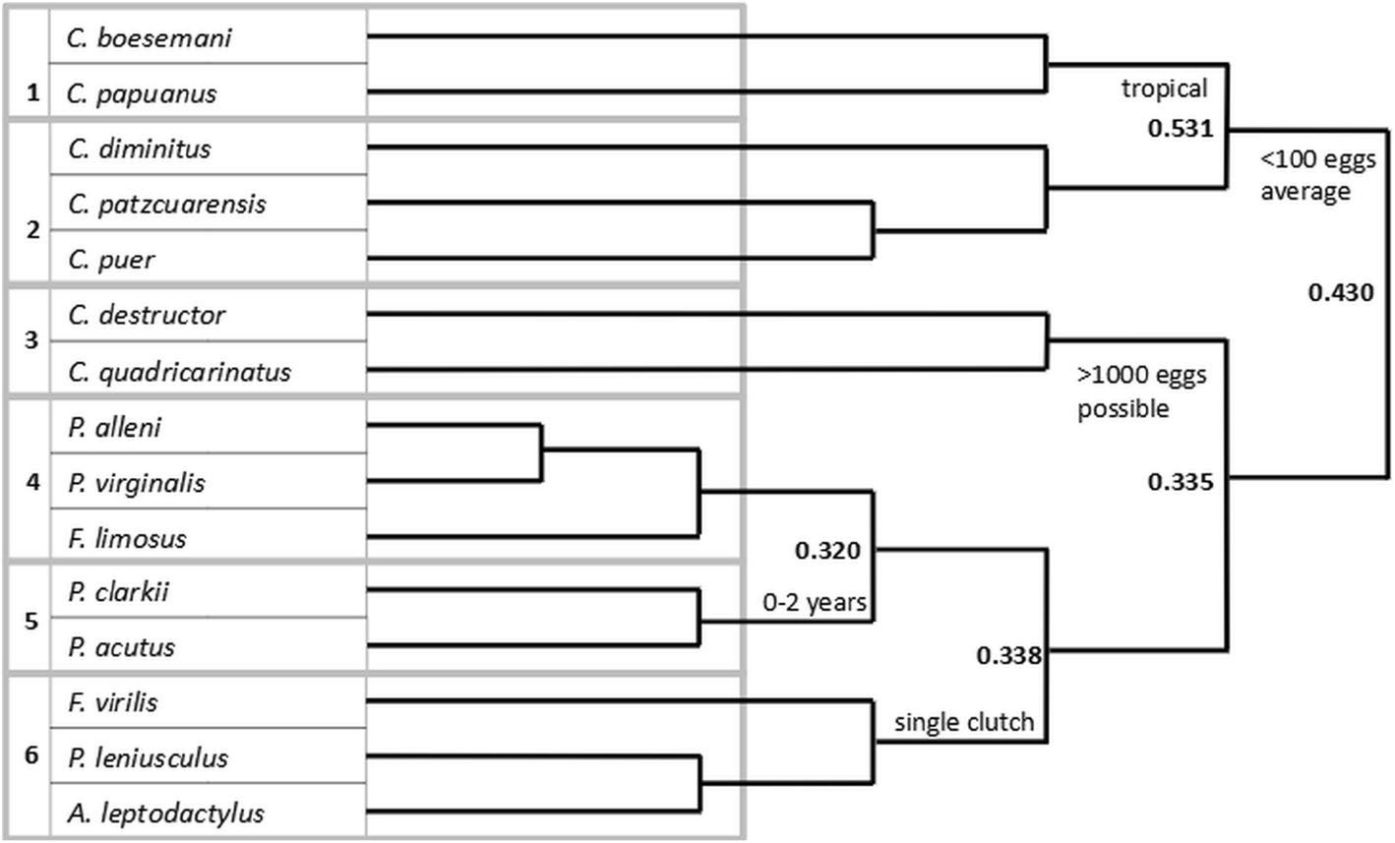
TWINSPAN cluster dendrogram with differentiating functional traits and eigenvalues indicated.

In the first two-way indicator species analysis (TWINSPAN) division all three *Cambarellus* spp. and both *Cherax boesemani* and *Cherax papuanus* were separated based on their low egg production trait (Fig. 4, Table 2). At the second division, the *Cambarellus* spp. were separated from *C. boesemani* and *C. papuanus* due to the tropical native range of both *Cherax* spp., contrasting to the temperate climate range of *Cambarellus* spp. Both *Cherax destructor* and *Cherax quadricarinatus* were grouped based on their potential to lay over 1000 eggs, where the eight remaining species produce over 100 eggs on average. Of these species, *A. leptodactylus, F. virilis* and *P. leniusculus* were grouped based on the production of a single clutch of eggs per year, while the other crayfish produce multiple clutches. Finally, *P. acutus* and *P. clarkii* formed their own group due to a shorter lifespan of up to 2 years, while *F. limosus*, *Procambarus alleni* and *Procambarus virginalis* in the last cluster can have a lifespan up to 3 to 4 years. Half of the clusters resulting from the TWINSPAN analysis include invasive species, whereas two groups consisted of exclusively invasive species. The eigenvalues associated with the divisions ranged from 0.320 to 0.531, indicating that the resulting groups were quite similar. While functional traits can have clear phylogenetic patterns, species from both genera *Procambarus* and *Cherax* can be found in multiple clusters. This shows that even within a crayfish genus traits can vary substantially. This is further supported by the inclusion of species from three different genera in cluster six.

**Table 2:** Functional traits and invasion category of 15 crayfish species present in the Netherlands in the wild or in aquaria.

Functional traits	Modalities	Species	*Astacus leptodactylus*	*Cambarellus diminutus*	*Cambarellus patzcuarensis*	*Cambarellus puer*	*Cherax boesemani*	*Cherax destructor*	*Cherax papuanus*	*Cherax quadricarinatus*	*Faxonius limosus*	*Faxonius virilis*	*Pacifastacus leniusculus*	*Procambarus acutus*	*Procambarus alleni*	*Procambarus clarkii*	*Procambarus virginalis*
Invasion category			C	B	B	B	B	B	B	B	E	D	C	D	B	E	B
Habitat flow preference	Lentic		1	1	1	1	1	1	1	1	1	1	1	1	1	1	1
	Lotic		1	0	0	0	0	1	0	1	1	1	1	1	1	1	1
Climate tolerance	Tropical		0	0	0	0	1	0	1	1	0	0	0	0	1	0	1
	Arid		0	0	0	0	0	1	0	1	0	1	0	0	0	1	0
	Temperate		1	1	1	1	0	1	0	0	1	1	1	1	1	1	1
	Cold		1	0	0	0	0	0	0	0	1	1	1	1	0	0	0
Salinity tolerance	0–5 g/L		1	1	1	1	1	1	1	1	1	1	1	1	1	1	1
	5–18 g/L		1	0	0	0	0	1	0	1	1	1	1	0	1	1	0
	18–30 g/L		1	0	0	0	0	1	0	0	0	0	0	0	0	0	0
Parthenogenetic			0	0	0	0	0	0	0	0	1	0	0	0	0	0	1
Nr. of clutches per year	1		1	0	0	0	0	0	0	0	0	1	1	0	0	0	0
	2		0	1	1	1	1	0	1	1	1	0	0	1	1	0	1
	3 +		0	0	0	0	0	1	0	0	0	0	0	0	0	1	0
Nr. of eggs	< 100 avg		0	1	1	1	1	0	1	0	0	0	0	0	0	0	0
	> 100 avg		1	0	0	0	0	0	0	0	1	1	1	1	1	1	1
	> 1000 possible		0	0	0	0	0	1	0	1	0	0	0	0	0	0	0
Growth rate	Slow		1	0	0	0	1	0	1	0	0	0	1	0	0	0	0
	Medium		0	1	1	1	0	0	0	1	1	1	0	0	1	0	1
	Fast		0	0	0	0	0	1	0	0	0	0	0	1	0	1	0
Migration distance	Short		0	1	1	1	1	0	1	0	0	1	1	0	0	0	0
	Medium		0	0	0	0	0	1	0	1	1	0	0	1	0	0	1
	Long		1	0	0	0	0	0	0	0	0	0	0	0	1	1	0
Moving over land			0	0	0	0	0	1	0	1	1	0	1	1	1	1	1
Body size (TLmm)	< 80 mm		0	1	1	1	0	0	0	0	0	0	0	0	0	0	0
	80–150 mm		1	0	0	0	1	1	1	0	1	1	0	1	1	1	1
	> 150 mm		0	0	0	0	0	0	0	1	0	0	1	0	0	0	0
Lifespan	0–2 y		0	1	1	1	0	0	0	0	0	0	0	1	0	1	0
	3–4 y		0	0	0	0	0	0	0	0	1	1	0	0	1	0	1
	5–6 y		0	0	0	0	1	1	1	1	0	0	0	0	0	0	0
	7 + y		1	0	0	0	0	0	0	0	0	0	1	0	0	0	0
Aggression	Low		0	1	1	1	0	0	1	0	1	0	0	0	0	0	0
	Medium		1	0	0	0	1	0	0	1	0	1	0	0	1	0	1
	High		0	0	0	0	0	1	0	0	0	0	1	1	0	1	0
Crayfish plague resistance			0	1	1	1	0	0	0	0	1	1	1	1	1	1	1

Literature sources for trait values can be found in Supplementary Tables S2 and S3 of the supplementary materials.

### Uncertainty of trait information

The uncertainty of trait information differs per species as well as per trait (Table 3), with an average uncertainty score across both species and traits of 9%. *Cherax boesemani* and *C. papuanus* showed the highest degree of uncertainty (35%). *Cambarellus diminitus* and *Cambarellus patzcuarensis* roughly had 20% uncertainty, while *Cambarellus puer* had about 12%. The functional traits of both aggression and growth rate showed highest uncertainty (about 20%), closely followed by the traits of migration distance and number of clutches per year (about 17%).

**Table 3:** Uncertainty calculated in percentage for each functional trait and crayfish species.

Species	Uncertainty (%)	Traits	Uncertainty (%)
*Astacus leptodactylus*	0	Aggression	20
*Cambarellus diminutus*	23	Body size	0
*Cambarellus patzcuarensis*	19	Crayfish plague resistance	10
*Cambarellus puer*	12	Growth rate	20
*Cherax boesemani*	35	Habitat flow preference	0
*Cherax destructor*	0	Lifespan	7
*Cherax papuanus*	35	Migration distance	17
*Cherax quadricarinatus*	0	Moving over land	3
*Faxonius limosus*	0	Native range	0
*Faxonius virilis*	0	Nr. of clutches per year	17
*Pacifastacus leniusculus*	0	Nr. of eggs	10
*Procambarus acutus*	8	Parthenogenetic	0
*Procambarus alleni*	4	Salinity tolerance	13
*Procambarus clarkii*	0	Average	9
*Procambarus virginalis*	0		
Average	9		

## Discussion

During the stages of invasion, different functional traits can contribute to invasive success of crayfish. Functional traits that separate successful from unsuccessful invaders were a medium egg count and temperate climate adaptation. Crayfish currently in the establishment stage of invasion were separated from fully invasive species by laying only a single clutch per year. The traits parthenogenesis and crayfish plague resistance, contrary to expectation, did not show a clear contribution to invasiveness. Hereafter, the role of functional traits in the invasion stages of establishment, spread, and fully invasive is discussed.

### Establishment stage

#### Environmental tolerances

In the establishment stage local climate becomes an important factor in survival. *Cherax boesemani* and *C. papuanus* were separated from the *Cambarellus* spp. based on their tropical native range (Fig. 4). All currently invasive crayfish are adapted to a temperate climate in their native range, most probably a requirement for invasive success in the Netherlands with a temperate ocean climate (Köppen-Geiger: Cfb). All invasive species, except *P. clarkii,* also occur in colder climates in their native range, further stressing that adaptation to cold periods can be very useful to survive European Atlantic winters. Larson & Olden^34^ reported niche shifts in *P. clarkii* and *P. leniusculus* with decreased mean maximum temperature and increased minimum temperature variance in comparison to their native range, suggesting that crayfish may survive different climatic condition in their invasive ranges. A separate study confirmed *P. clarkii*’s cold tolerance through experimental exposure to winter temperatures in a temperate climate^35^. In contrast, *C. quadricarinatus* did not survive these experimental conditions, which matches the expectations based on its tropical and arid climate native range. A mismatch between a species’ tolerances and environment means that survival is only potentially possible at locations exhibiting extraordinary environmental circumstances, as was the case where a population of *C. quadricarinatus* occurred in an oxbow lake in Slovenia with increased temperatures due to occurrence of hot water springs^36^.

#### High reproductive potential

Medium to high numbers of eggs contributes to higher invasive success as found in plants^37^, fish^38^ and gammarids^29^. The first TWINSPAN division excluded species with an average egg count lower than 100. Previous research also found high fecundity to be predictive of invasive success^32^, and clutch size most predictive for transitioning through stages from introduction to establishment to spread^23^. Yet, despite the high potential of egg numbers, *C. destructor* and *C. quadricarinatus* were currently not successful invaders. For *C. quadricarinatus* its climate tolerance could be the cause, however, *C. destructor* has been shown capable to survive under temperate climate winter temperatures^35^. Therefore, a different trait likely determines its lack of invasive success. Besides the number of eggs per clutch, the number of clutches produced also affects invasiveness: the most successful invaders *P. clarkii* and *F. limosus* are capable of laying more than a single clutch per year. Larson & Olden^32^ also reported the trait multiple annual reproductive events to be associated with invasive success, which supports our findings. Most crayfish species currently in the establishment and spread phases produce one clutch of eggs and are moderately successful. Although capable of producing three or more clutches, *C. destructor* is unsuccessful, suggesting it might lack other important traits.

### Spread and dispersal stage

#### Migration and moving over land

When colonising new freshwater habitats, migration capacity is an important determinant of spread^39^. However, migration capacity did not determine any division between successful and unsuccessful invaders in the TWINSPAN analysis. Although long migration distances were only found in clusters that included invasive crayfish and five of the nine unsuccessful species have short migration distances, the short migration capacity of *F. virilis* and *P. leniusculus* implies that this trait did not clearly contribute to the invasive potential. Moving over land, while less effective than aquatic dispersal, could greatly increase dispersal in crayfish^40^. While not all currently invasive crayfish can move over land, those that do vary in invasive success. The contribution of dispersal traits to invasiveness appears in our data to be outweighed by other traits. In addition, human mediated propagule pressure, such as repeated introduction through pet releases, could also be important during the spread stage by supporting dispersal^20,22^.

### Fully invasive stage

#### Large size

A small size can be disadvantageous for crayfish due to increased predation risk and lower competitive capacity^41^ but did not result in cluster divisions. In contrast, other authors found invasive crayfish species to be characterised by maximum size^32^. Interestingly, the number of eggs produced appeared an explanatory trait causing grouping and is generally positively related to maximum size^42–44^. The *Cambarellus* spp. separated based on low egg count in the first division are also the smallest species considered. *Cherax destructor* and *C. quadricarinatus* with the highest potential egg counts are also some of the largest species taken into account. It is possible that potential effects of size on invasive success are masked by the impact of egg count. Zeng et al.^23^ found clutch size a very important invasive trait when scaled to carapace length, supporting this observation.

#### Aggression

The level of aggression displayed by current invaders differs from low to high and consequently this trait did not explain invasion success. In contrast, other studies using chelae size as indicator for dominance and competitive ability did find this trait indicative of extraregional invasive success (species that have invaded another continent or crossed major drainage boundaries in their native continent)^32^ and in the transition to the introduction stage^23^. While *F. limosus* is found in lotic waters it prefers to live in ponds and lakes^45,46^. However, it is less common in the Dutch deep lakes and ponds then its preferences would suggest. This distribution constraint could be the effect of competitive exclusion by the more aggressive *P. clarkii*, which is mainly found in these waters, forcing *F. limosus* and other species into less optimal water types^16^. Although *P. clarkii* has a high level of aggression and *F. limosus* a low level of aggression, both are very successful invaders, indicating other traits might offer alternative strategies for high invasiveness.

#### Crayfish plague

In Europe, the introduction of the crayfish plague, *Aphanomyces astaci*, from North America has decimated many indigenous crayfish species, which are much more susceptible to this pathogen than North American species^47^. Although sensitive to this plague, *A. leptodactylus* is interestingly clustered together with two North American species that are known carriers^48^. In fact, *P. leniusculus* was responsible for four recorded mass mortalities of the indigenous *Austropotamobius pallipes* in France^49^. This clustering likely results from the shared evolutionary history between *A. leptodactylus* and *P. leniusculus*, both belonging to the family *Astacidae*^50^. A phylogenetic signal such as this could outweigh the importance of other trait dissimilarities between species during clustering, which might be the case with crayfish plague resistance. In the Netherlands, *A. leptodactylus* was found almost exclusively in fast flowing and brackish to saline waters, even though it is also adapted to lentic habitats^51^. *Astacus leptodactylus* only occurs near North American species at a few locations and is mainly found in water types where those species are absent. It could be excluded from many water types by presence of plague carriers, while its high salinity tolerance allows it to survive where the other species cannot. Similarly, *C. destructor*, despite possessing several beneficial invasive traits is susceptible to the crayfish plague which might prevent successful establishment^48^. With the growing presence of crayfish from North America, it seems likely that plague resistance is mandatory to reach the final full invasive stage in the Netherlands.

### High invasive success and functional strategies

Crayfish species predicted to become extraregional invaders by Larson & Olden^32^ were *F. virilis, P. acutus* and *P. clarkii*, while the species with the highest FI-ISK scores were *P. clarkii*, *P. leniusculus* and *F. limosus*^33^. All of these species currently occur in the Netherlands. *Faxonius virilis*, *P. leniusculus*, and *P. acutus* were first reported in 2004, 2005, and 2007 respectively, giving them roughly the same time frame for their invasions. However, *P. leniusculus* (stage establishment) showed a far smaller geographical distribution compared to both other species (stage spread). This difference between prediction and current invasive status might be caused by the importance given to import for aquaculture in the FI-ISK tool, while this study is focused on the traits of invasion stages that follow after introduction. The other highest risk species, *F. limosus* and *P. clarkii*, are fully invasive in the Netherlands, but differ in their traits and invasiveness trajectory. Although more widely distributed, *F. limosus* is most frequently found in small linear lentic waters and slow flowing waters, which *P. clarkii* rarely inhabits. This conforms to the difference in aggression between these species; *P. clarkii* being more aggressive could lead to competitive exclusion of *F. limosus*. While *P. clarkii*’s higher migration capacity should bring advantage over *F. limosus* in occupying new areas, the latter’s widespread occurrence suggests that parthenogenetic reproduction might counteract its lower migration capability. While *P. clarkii* can theoretically produce more clutches of eggs in a year, it requires individuals from both sexes to do so. In contrast, *F. limosus* can potentially reproduce with a single individual to establish a new population, increasing its invasiveness. Although facultative parthenogenesis in *F. limosus* has been experimentally confirmed^52^, its frequency of occurrence in wild populations is uncertain. And although not reported in the literature, parthenogenesis cannot be excluded in *P. clarkii*. Furthermore, *P. clarkii* has been shown to survive Dutch winter temperatures and for *F. limosus* a cold climate is part of its native range. This might explain the different distributions of these species in the Netherlands, with *P. clarkii* surviving in deeper waters where it can survive winter conditions and *F. limosus* being capable of surviving in different water types due to its inherent cold climate tolerance. Ultimately, these two species have a different combination of functional traits leading to alternative strategies that both are successful in becoming fully invasive.

### Who is the next invasive species?

FI-ISK invasion risk predictions available for crayfish present in the Dutch aquarium trade range from medium (*C. puer*, *C. quadricarinatus*, *P. virginalis*) to high (*C. destructor*)^33^. *Cambarellus puer* has a small size and produces lower numbers of eggs, resulting in a low risk of invasion for the Netherlands. Due to widespread presence of North American species potentially carrying crayfish plague, the susceptible *C. quadricarinatus* and *C. destructor* seem unlikely candidates for widespread invasion in the Netherlands. This notion is supported by the apparent mismatch between their climate tolerance and the Dutch climate. The combination of these traits also excludes the other *Cherax* spp. in this study. Although not currently found in the wild in the Netherlands, both *P. virginalis* and *P. alleni* are clustered with the very successful invader *F. limosus*, suggesting they also possess those traits necessary to become invasive. However, these *Procambarus* species and *F. limosus* differ in their climate tolerance too, with only the latter being adapted to a colder climate. *Procambarus virginalis* is adapted to a humid subtropical and temperate climate but can survive water temperatures lower than 8 °C and even below 2 °C for several weeks^53^. Here, climate change could make a difference in the near future. Furthermore, both *F. limosus* and *P. virginalis* can reproduce parthonegetically, increasing chances of establishing populations^54^, but *P. alleni* has not been reported to be capable of this. Tricarico et al.^33^ predicted *P. virginalis*’ invasion risk in Italy to be medium, but caution against its release into the wild. Chucholl^55^ found that higher human population density and availability of lentic water can increase the likelihood of *P. virginalis* releases. *Procambarus virginalis* has established wild populations in Germany, Italy, and Slovakia^17,51,56^ and has already been recorded once in the Netherlands^57^. However, no subsequent sightings have been reported. In contrast, no established populations of *P. alleni* have currently been identified in Europe^17^ and no sightings have been reported in the Netherlands. Due to availability in the Dutch aquarium trade, its functional traits with high invasive potential and invasion history in Europe, *P. virginalis* is most likely to become the next successful invader in the Netherlands.

Early detection of *P. virginalis* introductions is highly important to make informed management decisions, therefore we recommend preventative monitoring. A potential tool for this is environmental DNA monitoring, which has already been successfully applied for targeted detection of *P. virginalis* in several lakes and rivers in Germany^58^. In Norway, eDNA detection of invasive crayfish and the spread of the crayfish plague worked so well that it has been adopted in multiple surveillance programmes^59^. Although not yet suitable for assessing species abundance, presence/absence data can reliably be obtained in Dutch water systems using high throughput sequencing techniques and potentially be gathered through non-targeted monitoring^60^. An advantage of such approach would possibly provide early detection of *P. virginalis* or other invasive crayfish during routine eDNA sampling efforts of Dutch water authorities. Additionally, targeted monitoring could be informed by predictions of invasive crayfish distribution through ecological niche models (ENM) and species distribution models (SDM)^61^. Examples of such predictions are ENMs composed for *C. destructor*, *P. leniusculus* and *P. clarkii*^62^ and SDMs developed for *P. leniusculus* and *P. carkii*^63^, which show their potential geographic distributions currently and under the impacts of climate change, respectively.

### Other conditions affecting invasiveness

Several factors determining the resistance of native communities to non-indigenous species, such as predation and competition, increase with native species richness. Disturbance of aquatic habitats by human activities often reduces local biodiversity, and consequently the resistance of native communities to invasion^64^. Colautti et al.^37^ found that increased environmental disturbance and resource availability both significantly contributed to successful invasions. Furthermore, an increase in number of people does increase the chance onto a higher propagule pressure and at the same an increase in environmental pressure providing more opportunity for invasive species. The only crayfish native to the Netherlands, *Astacus astacus*, has almost gone extinct due to the crayfish plague, opening up a niche space for non-indigenous species. Such niche vacancies can also occur due to anthropogenic environmental influences, e.g., Früh et al.^65^ reported that German river and stream sites with invasive species were significantly more degraded than sites without non-indigenous species. In the Netherlands, many waters have been thoroughly altered and many are created by humans. Anthropogenic disturbance might have facilitated invasiveness; the distribution of *P. clarkii*, *F. virilis* and *P. acutus* is centred around the largest cities in the Netherlands and nearby densely populated areas, where anthropogenic disturbances are intense.

Evolutionary adaptation is related to time of arrival. Whitney and Gabler^26^ reported 82 evolutionary changes in 38 species of plants and animals between their native and introduced ranges. Each trait considered was adapted in several species, showing that changes in invasive traits are relatively common. Additionally, some changes occurred within 20 years, a much shorter time-span than *F. limosus*, *A. leptodactylus* and *P. clarkii* are present in the Netherlands. Such rapid evolutionary change could be caused by adaptive phenotypic plasticity, where invasive populations experience directional selection resulting in enhanced fitness^66^. Niche shifts observed in crayfish regarding climate adaptation^34^ might be an example of this process and could have contributed to the current invasive success of *F. limosus* and *P. clarkii*.

## Conclusion

While not all traits studied appeared to contribute to crayfish invasion in the Netherlands, more successful invaders were distinguished by several traits. During establishment, being adapted to a temperate climate is mandatory for survival. Not having a low egg count is required for establishing a population, while producing more than a single clutch per year increases invasive potential. The importance of these traits for crayfish invasiveness is confirmed by literature. Some traits have less straightforward contributions to crayfish invasiveness: besides a temperate climate tolerance, being adapted to a cold climate can improve winter survival. A high level of aggression as seen in *P. clarkii* might lead to exclusion of competitors and increase invasiveness. Parthenogenetic reproduction potentially contributed to *F. limosus* passing through stages establishment and spread, and resistance to the crayfish plague has become another important invasion trait due to increased presence of North American crayfish species. Since specific combinations of the traits mentioned above lead to successful invaders, functional trait analysis can be a valuable tool in predicting invasiveness of non-indigenous crayfish species. Using these traits as a descriptive prediction tool, *P. virginalis* is predicted to become a future successful invader in the Netherlands. This prediction conforms to predictions for *P. virginalis* from previous risk assessment studies.

## Methods

The Dutch food and consumer product safety authority (NVWA) does not officially register crayfish aquaculture companies and records of crayfish trade and import are not digitally available. To determine which nonindigenous crayfish are present in the Netherlands, a web search was performed (20-06-2018) for crayfish being sold. The search terms ‘aquarium store crayfish’ and ‘buy aquarium crayfish’ were used (in Dutch) in two separate searches on www.google.com, resulting in 16 websites offering 19 species of live crayfish in the Netherlands (Supplementary Table S1 online). Species only sold on a single website were excluded due to lower potential for introduction into the wild. Per genus, the three species available on most websites were selected for further research. For *C. boesemani* several colour morphs were available for sale, which were treated as a single species. Additionally, *C. destructor* was selected since it is a habitat generalist with a widespread native range^51^, and it has already established populations in Europe; Italy and Spain^67^. The search resulted in a list of ten species: *C. diminitus*, *C. patzcuerensis*, *C. puer*, *C. quadricarinatus*, *C. boesemani*, *C. destructor*, *C. papuanus*, *P. alleni, P. clarkii* and *P. virginalis*. Species with verified sightings in the wild in the Netherlands, *A. leptodactylus*, *F. limosus*, *F. virilis*, *P. leniusculus, P. acutus* and *P. clarkii*, were obtained from a dataset from Stichting European Invertebrate Survey (EIS)^68^. This resulted in a total of fifteen crayfish species for further analysis (Table 2). *Procambarus virginalis* and *C. quadricarinatus* have been reported only once in the Netherlands^21^ and were for the purpose of this study not considered to occur in the wild.

### Distribution

For the six species in the EIS dataset the number of occurrences (records at 3251 different sites in total) per year was plotted from the first introduction or documented sighting until 2015. Based on this data it appeared that crayfish records increased dramatically around 2007 (Fig. 1). This was due to a strong increase in targeted monitoring by water authorities. Therefore, the more extensive and reliable data from 2007 to 2015 were used for our analyses. Each species was assigned a category of invasive success (Table 2) according to the classification scheme by Blackburn et al.^24^, simplified by combining subcategories (B1, B2, B3) into main categories (B). This classification was performed based on three factors. First, the presence of juveniles or berried females (carrying eggs) as evidence for self-sustaining populations. Second, the number of sites where they were encountered classified into the categories B = 0, C =  > 10, D =  > 100 and E =  > 1000. Thirdly, the spatial scale of the distribution range of each crayfish species in the Netherlands (Fig. 2) classified as C = local, D = regional and E = national.

### Water types

The occurrences of the six successful invasive species were plotted on a map of the Netherlands in ArcMap 10.2.2 (ESRI, Redlands, CA) (Fig. 2a,b). The coordinates of these records were matched to a NLtop10 based aquatic map^69^, with the closest waterbody within a 200 m radius being assigned to the crayfish sightings. When available within the aquatic map, a Water Framework Directive (WFD) water type was determined as a proxy for environmental conditions, resulting in 1888 records at 615 different sites. Water types were then grouped together in main categories based on their similarity in environmental conditions: deep lakes and ponds, small lentic linear waters, slow flowing linear waters, large lentic linear waters, shallow lakes and ponds, weak brackish to saline waters and fast flowing waters. For each category the number of sites was counted per species.

### Functional traits

Functional traits were selected based on the filters described in the invasion framework and commonly used in invasion literature for the stages establishment, spread and fully invasive^24–26^. The traits included for analysis were: habitat flow preference^23,32,33^, climate preference^33^, salinity preference^33^, parthenogenesis, number of clutches per year^23,32,33^, number of eggs per clutch^23,32,33^, growth rate, migration distance^33^, migration over land^33^, maximum body size^23,32^, maximum age, level of aggression^23,32,33^, and resistance against the crayfish plague (Table 2). The traits generalist diet, short generation time and ability to escape or survive natural enemies from Table 1 were excluded for varying reasons. For example, crayfish have a generalist and flexible diet resulting in high similarity between all species included. For the selected traits, information was obtained through literature research; species were assigned trait modalities based on data from scientific papers (Supplementary Tables S2 and S3 online). For each trait modality a species scored either 1 or 0, with the possibility to score 1 in multiple modalities for some traits. When trait information for a species was unavailable from literature its modalities were assigned through expert judgement, mostly based on confirmed traits of species within the same genus. For the traits growth rate, migration and aggression the information obtained from literature varied in expression, complicating the definition of clear trait modalities. Here, comparative studies between species were used when possible to assign crayfish into generic categories of ‘low’, ‘medium’ and ‘high’. The Köppen-Geiger climate classifications^70^ of each species’ native range (Supplementary Table S2 online) were used to assign climate preference. *Procambarus virginalis* is a species that originates from the aquarium trade^71^ and has no natural home range, thus the native range of its closest relative, *Procambarus fallax*, was used. No scientific literature was available on the traits growth and aggression for the species *C. boesemani* and *C. papuanus*. Therefore, information from aquarium websites was included.

Expert judgement has previously been used to assign trait values to aquatic invertebrates^72,73^ and plants. In the latter it has been found to correlate strongly with experimentally derived trait information^74^. However, it does introduce a degree of uncertainty in the species by traits table. To address this issue uncertainty values were calculated (Table 3). Traits taken from literature were scored as 1, traits assigned through expert judgement scored 0.5 and traits based on aquarium websites received a score of 0.1. For each functional trait these scores were added up, divided by the total number of species, multiplied by and subtracted from 100 to obtain percentage-based uncertainty values. The same calculation was performed for each species.

### Statistical analysis

Due to the binary nature of our species by traits matrix, the relatively small number of species and expected hierarchy in the data, TWINSPAN hierarchical cluster analysis^75^ was chosen to determine the relationship between crayfish traits and their invasive success. TWINSPAN analysis has often been applied on plants to identify functional groups based on their traits^76,77^. For example, TWINSPAN was applied to classify plant communities into multiple stages of succession using functional traits^78^. The TWINSPAN method constructs hierarchically ordered two-way tables in multiple steps^79,80^. First, the dataset is split into two subsets roughly in the middle of the primary axis of a correspondence analysis (CA). This is repeated on each subset for further divisions to obtain a divisive hierarchy of the data and convert it into an ordering. Usually this is done on a sites by species matrix with ordering of the data by site, however, we used a species by traits matrix and first ordered the data based on species identity. Secondly, the other side of the data matrix is analysed in a similar manner but includes the constructing of attributes based on the splits in the first divisive hierarchy. In our analysis this step consisted of constructing these attributes for the traits in our matrix and using these to divide the dataset into groups based on functional traits. Finally, a two-way ordered table is created from the previous two steps, showing hierarchical clusters and indicators for each division. In our case the resulting divisions in clusters of crayfish species were indicated by specific functional traits. We performed TWINSPAN analysis in WinTwins (Version 2.3 for Windows^79^) using a minimum group size of four for division and weighting all traits according to their uncertainty (Table 3). A dendrogram was constructed to visualize the resulting clusters and divisions (Fig. 4). The dissimilarity between clusters was quantified in eigenvalues from low (0) to high (1) at each split.

## Supplementary Information

Supplementary Information
