# Supplementary material for: Functional traits explain crayfish invasive success in the Netherlands
Source: Sci Rep. 2021 Feb 2;11:2772. doi: 10.1038/s41598-021-82302-4 (PMC7854663; doi:10.1038/s41598-021-82302-4)
Supplement: Supplementary file 1 — Supplementary Information [file 41598_2021_82302_MOESM1_ESM.pdf]

Supplementary Information

Functional traits explain crayfish invasive success in the Netherlands

1st author*: Tiedo van Kuijk (1,2)
Email: tiedo.vankuijk@naturalis.nl
telephone number: + 31-(0)71 751 96 00
Address: P.O. Box 9517, 2300 RA Leiden, The Netherlands
ORCID: 0000-0002-6585-5558

2nd author: Jacobus C. Biesmeijer (1,3)
Email: koos.biesmeijer@naturalis.nl
Address: P.O. Box 9517, 2300 RA Leiden, The Netherlands
ORCID: 0000-0003-0328-0573

3d author: Berry B. van der Hoorn (1)
Email: berry.vanderhoorn@naturalis.nl
Address: P.O. Box 9517, 2300 RA Leiden, The Netherlands

Final (4th) author: Piet F. M. Verdonschot (2,4)
Email: piet.verdonschot@wur.nl
Address: P.O. Box 47, 6700 AA Wageningen, The Netherlands
ORCID: 0000-0002-4126-7452

1) Naturalis Biodiversity Center, Leiden, Netherlands
2) Institute for Biodiversity and Ecosystem Dynamics, University of Amsterdam, Amsterdam, The Netherlands
3) Institute of Environmental Sciences, Leiden University, Leiden, The Netherlands
4) Wageningen Environmental Research, Wageningen UR, Wageningen, The Netherlands

Supplementary Tables

Supplementary Table S1

	Websites																Total
Species	1	2	3	4	5	6	7	8	9	10	11	12	13	14	15	16	
A. leptodactylus	-	-	-	x	-	-	-	-	-	-	-	-	-	-	-	-	1
C. diminutus	x	x	x	x	-	x	-	-	-	-	x	-	-	-	-	x	7
C. patzcuarensis	x	x	-	x	x	x	x	x	x	-	x	x	x	-	x	x	13
C. puer	-	x	-	x	-	x	-	-	-	-	-	-	-	-	-	-	3
C. texanus	-	x	-	-	-	x	-	-	-	-	-	-	-	-	-	-	2
C. pecknyi	-	-	-	-	-	-	-	x	-	-	-	-	x	-	-	-	2
C. quadricarinatus	-	-	-	-	-	-	-	x	-	-	x	-	-	x	-	-	3
C. boesemani (Blue Moon)	-	-	-	x	-	-	-	x	-	-	-	-	-	-	-	-	2
C. boesemani (Hoa Creek)	-	-	-	x	-	-	-	x	-	-	-	-	-	-	-	-	2
C. boesemani (Red Brick)	-	-	-	x	-	-	-	-	-	-	-	-	-	-	-	-	1
C. destructor	x	-	-	x	-	-	-	-	-	-	-	-	-	-	-	-	2
C. holthuisi	-	-	-	-	-	-	-	x	-	-	-	-	-	-	-	-	1
C. papuanus	-	x	-	x	-	x	-	x	-	-	-	-	x	-	-	-	5
O. limosus	-	-	-	x	-	-	-	-	-	-	-	-	-	-	-	-	1
P. clarkii	x	-	-	x	-	x	-	x	-	-	-	-	-	x	-	x	6
P. alleni	x	x	x	x	-	x	x	x	-	x	-	-	x	-	-	-	9
P. virginalis	x	-	-	-	-	-	-	-	-	-	-	x	-	x	-	-	3
P. acutus	-	-	-	x	-	-	-	-	-	-	-	-	-	-	-	-	1
P. troglodites	-	-	-	-	-	-	-	-	-	-	-	x	-	-	-	-	1

1 marktplaats.nl, 2 heevis.nl, 3 aquafizz.nl, 4 aquariaveldhuis.nl, 5 aquariumvissenwinkel.nl, 6 gdaquarium.nl, 7 hornbach.nl, 8 chrisgarnalenshop.nl, 9 matavi.nl, 10 aquariumkoning.nl, 11 aquariumcentrum.nl, 12 dierenhandelexotica.nl, 13 ruinemans.com, 14 dierenspeciaalzaakxxl.com, 15 koicompagnie.nl, 16 detropischeviskwekerij.nl

Supplementary Table S1. List of crayfish species found for sale online in the Netherlands on 20-06-2018. Website names can be found at the bottom.

Supplementary Table S2

**Species** **Traits**	Habitat flow preference	Salinity tolerance	Parthenogenetic	Nr. of clutches per year	Nr. of eggs	Growth rate	Migration distance	Moving over land	Lifespan	Body size (TLmm)	Aggression	Crayfish plague resistance
Astacus leptodactylus	13	8,13,32	-	39	15,39	5	15	-	13,15,28	38	5,15	5,15,25
Cambarellus diminutus	7,24	a	-	c	e	g	24	-	13,37	24,51	n	q
Cambarellus patzcuarensis	7	a	-	c	e	12	j	-	13,37	12,51	n	25,34,36
Cambarellus puer	7	64	-	51,60	37,51	g	j	-	13,37	51	32	q
Cherax boesemani	16	b	-	d	16	h	k	-	m	16	o	r
Cherax destructor	13	20,59	-	13	13, 32	13	13	62	32,58	47	17, 65	25
Cherax papuanus	29	b	-	d	f	h	k	-	m	30	p	25
Cherax quadricarinatus	56	22	-	13	11,13,22,23	1,11	61	46	11,13	46	11	25
Faxonius limosus	7,49	9	31	40	3,15,23	40,49	15,61	49	49	31	13,15	13,15,25,33
Faxonius virilis	2,6,7	20	-	41	3,21,35	21	6	-	13,32	6,32,49	2,5	13,25,33
Pacifastacus leniusculus	7	8	-	52	15	5	15,61	48	13,15,28,32	42	13,15	13,15,25,33
Procambarus acutus	7	35	-	50	18,19	19	i	l	35	18	19,44	25,33
Procambarus alleni	7	53	-	d	26	26	58	57	54	14,26	26	25
Procambarus clarkii	7	63	-	32	3,4,23	4,19	4,13,32	45,57	13,32	43	13,43	4,13,25,33
Procambarus virginalis	7	62	10,13	27	13,23,26	10,23,26	58	58	13,27,28	14,26,27	10,13	13,25

Supplementary Table S2 (continued)

**Species**	Native range
Astacus leptodactylus	(13) Tributaries of the Caspian Sea (Wolga, Ural) and the tributaries of the Ob River
Cambarellus diminutus	(7,24) Restricted ranges in southeastern Mississippi and southwestern Alabama
Cambarellus patzcuarensis	(7) Lago de Patzcuaro, Michoadin, Mexico
Cambarellus puer	(7) Texas, Arkansas, Mississippi and Tennessee.
Cherax boesemani	(16) Ajamaru Lakes, Vogelkop peninsula, West New Guinea
Cherax destructor	(13) Large parts of Australia, mainly the eastern half
Cherax papuanus	(29) Lake Kutubu, Papua New Guinea
Cherax quadricarinatus	(11) Queensland and the Northern Territory, northern Cape York, west of the Gulf and New Guinea.
Faxonius limosus	(7) The Atlantic watershed from Maine to the lower James River in Virginia.
Faxonius virilis	(7) Saskatchewan to Ontario, Canada, Montana and Wyoming to New York and southwestern Maine
Pacifastacus leniusculus	(7) British Columbia, California, Idaho, Nevada, Oregon, and Washington
Procambarus acutus	(7) Maine to Georgia, Florida to Texas, Minnesota to Ohio, southwestern Texas and northern Mexico
Procambarus alleni	(7) East of St. Johns River and all of peninsular Florida south of Levy and Marion counties
Procambarus clarkii	(7) Northern Mexico to Escambia County, Florida, and north to southern Illinois
Procambarus virginalis	(7) Tributaries of the Satilla River, Georgia, southward through peninsular Florida

Supplementary Table S2. Overview of trait information sources for Table 2. Numbers and letters refer to literature references and expert judgement, respectively, in Supplementary Table S3. Traits not present in a species are marked -.

Supplementary Table S3

Literature references

1	Austin, C. M. Preliminary pond production of the red claw crayfish, Cherax quadricarinatus, in the Central United States. *J.Appl. Aquac.*, **1,** 93-102 (1993).
2	Bovbjerg, R. V. Ecological isolation and competitive exclusion in two crayfish (Orconectes virilis and Orconectes immunis). *Ecology*, **51,** 225-236 (1970).
3	Corey, S. Comparative fecundity of four species of crayfish in southwestern Ontario, Canada (Decapoda, Astacidea). *Crustaceana*, **52,** 276-286 (1987).
4	Gherardi, F. Crayfish invading Europe: the case study of Procambarus clarkii. *Mar. Freshw. Behav. Physiol.*, **39,** 175-191 (2006).
5	Harlıoğlu, M. M. A comparison of the growth and survival of two freshwater crayfish species, Astacus leptodactylus Eschscholtz and Pacifastacus leniusculus (Dana), under different temperature and density regimes. *Aquac. Int.*, **17,** 31-43 (2009).
6	Hazlett, B., Rittschof, D., & Rubenstein, D. Behavioral biology of the crayfish Orconectes virilis I. Home range. *Am. Midl. Nat.*, **92,** 301-319 (1974).
7	Hobbs Jr, H. H. A checklist of the North and Middle American crayfishes (Decapoda: Astacidae and Cambaridae). *Smithsonian Contrib. Zool.*, **166,** (1974).
8	Holdich, D. M., Harlioğlu, M. M., & Firkins, I. Salinity Adaptations of Crayfish in British Waters with Particular Reference toAustropotamobius pallipes, Astacus leptodactylusandPacifastacus leniusculus. *Estuar. Coast. Shelf Sci.*, **44,** 147-154 (1997).
9	Jaszczołt, J., & Szaniawska, A. The spiny-cheek crayfish Orconectes limosus (Rafinesque, 1817) as an inhabitant of the Baltic Sea—experimental evidences for its invasion of brackish waters. *Oceanol. Hydrobiol. Stud.*, **40,** 52-60 (2011).
10	Jimenez, S. A., & Faulkes, Z. Can the parthenogenetic marbled crayfish Marmorkrebs compete with other crayfish species in fights?. *J. Ethol.*, **29,** 115-120 (2011).
11	Jones, C. *The biology and aquaculture potential of the tropical freshwater crayfish, Cherax quadricarinatus.* (Department of Primary Industries Queensland, 1990).
12	Karadal, O., & Türkmen, G. Growth Performance and Substrate Preference of Juvenile Mexican Dwarf Orange Crayfish (Cambarellus patzcuarensis) in Different Substrate Types. *J. Limnol. Freshw. Fish. Res.*, **3,** 167-173 (2017).
13	*Freshwater crayfish: A global overview.* (ed. Kawai, T., Faulkes, Z., & Scholtz, G.) (CRC Press 2015).
14	Klassen, J. A., Gawlik, D. E., & Botson, B. A. Length–weight and length–length relationships for common fish and crayfish species in the Everglades, Florida, USA. *J. Appl. Ichthyol.*, **30,** 564-566 (2014).
15	Lozán, J. L. On the threat to the European crayfish: A contribution with the study of the activity behaviour of four crayfish species (Decapoda: Astacidae). *Limnologica*, **30,** 156-161 (2000).
16	Lukhaup, C., & Pekny, R. Cherax (Astaconephrops) boesemani, a new species of crayfish (Crustacea: Decapoda: Parastacidae) from the centre of the Vogelkop Peninsula in Irian Jaya (West New Guinea), Indonesia. *Zoologische Mededelingen*, **82,** 331-340 (2008).
17	Lynas, J., Storey, A. W., & Knott, B. Aggressive interactions between three species of freshwater crayfish of the genus Cherax (Decapoda: Parastacidae). *Mar. Freshw. Behav. Physiol.*, **40,** 105-116 (2007).
18	Mazlum, Y., & Eversole, A. G. Observations on the life cycle of Procambarus acutus acutus in South Carolina culture ponds. *Aquaculture*, **238,** 249-261 (2004).
19	Mazlum, Y., & Eversole, A. G. Growth and survival of Procambarus acutus acutus (Girard, 1852) and P. clarkii (Girard, 1852) in competitive settings. *Aquac. Res.*, **36,** 537-545 (2005).

20	Mills, B. J., & Geddes, M. C. Salinty tolerance and osmoregulation of the Australian freshwater crayfish Cherax destructor Clark (Decapoda: Parastacidae). *Mar*. *Freshw*. *Res*., **31,** 667-676 (1980).
21	Momot, W. T. Population dynamics and productivity of the crayfish, Orconectes virilis, in a marl lake. *Am. Midl. Nat.*, **78,** 55-81 (1967).
22	Ruscoe, I. *Redclaw crayfish aquaculture (Cherax quadricarinatus)* (Department of Primary Industry, Fisheries and Mines, Northern Territory Government 2002).
23	Seitz, R., Vilpoux, K., Hopp, U., Harzsch, S., & Maier, G. Ontogeny of the Marmorkrebs (marbled crayfish): a parthenogenetic crayfish with unknown origin and phylogenetic position. *J. Exp. Zoolog. Part A Comp. Exp. Biol.*, **303,** 393-405 (2005).
24	Stites, A., Taylor, C. A., & Schuster, G. A. *Status Survey for three rare Alabama crayfishes: Cambarellus diminutus, Fallicambrus burrisi, and Procambarus lagniappe.* (Prairie Research Institute, University of Illinois 2014).
25	Svoboda, J., Mrugała, A., Kozubíková-Balcarová, E., & Petrusek, A. Hosts and transmission of the crayfish plague pathogen Aphanomyces astaci: a review. *J. Fish. Dis.*, **40,** 127-140 (2017).
26	Vanarman, P. G. *Biology and Ecology of Epigean Crayfish That Inhabit Everglades Environments Procambarus alleni (Faxon) and Procambarus fallax (Hagen)* (Nova Southeastern University 2003).
27	Vogt, G. Marmorkrebs: natural crayfish clone as emerging model for various biological disciplines. *J. Biosci.*, **36,** 377-382 (2011).
28	Vogt, G. Ageing and longevity in the Decapoda (Crustacea): a review. *Zool. Anz.*, **251,** 1-25 (2012).
29	Imbun, B. Y., & Smith, P. T. Recent developments at Lake Kutubu: impacts on the fish and the foe. *Fishes Sahul*, **32,** 1224-1238 (2018).
30	Smith, P. T., Imbun, B. Y., & Duarte, F. P. Impacts of a Fish Kill at Lake Kutubu, Papua New Guinea 1. *Pac. Sci.*, **70,** 21-34 (2016).
31	Buřič, M., Kouba, A., & Kozak, P. Reproductive plasticity in freshwater invader: from long-term sperm storage to parthenogenesis. *PloS one*, **8,** e77597; https://doi.org/10.1371/journal.pone.0077597 (2013).
32	Reynolds, J., & Souty-Grosset, C. *Management of freshwater biodiversity: crayfish as bioindicators.* (Cambridge University Press. 2011).
33	Tilmans, M., *et al.* Survey of the crayfish plague pathogen presence in the Netherlands reveals a new Aphanomyces astaci carrier. *J. Invertebr. Pathol.*, **120,** 74-79 (2014).
34	Mrugała, A. *et al.* Trade of ornamental crayfish in Europe as a possible introduction pathway for important crustacean diseases: crayfish plague and white spot syndrome. *Biol*. *Invasions*, **17,** 1313-1326 (2015).
35	Rogowski, D. L., Sitko, S., & Bonar, S. A. Optimising control of invasive crayfish using life-history information. *Freshw*. *Biol*., **58,** 1279-1291 (2013).
36	Gál, B., Kuříková, P., Bláha, M., Kouba, A., & Patoka, J. Cambarellus patzcuarensis in Hungary: The first dwarf crayfish established outside of North America. *Biologia*, **72,** 1529-1532 (2017).
37	Alvarez, F., & Villalobos, J. L. *The crayfish of middle America in Freshwater crayfish. A global overview* (ed. Kawai, T., Faulkes, Z., & Scholtz, G.) 448-462 (CRC Press 2016).
38	Balık, İ., Özkök, E., & Özkök, R. Catch per unit effort and size composition of crayfish, Astacus leptodactylus Eschscholtz 1823, in Lake Iznik. *Asian-australas. J. Anim. Sci.*, **15,** 884-889 (2002).
39	Berber, S., & Mazlum, Y. Reproductive efficiency of the narrow-clawed crayfish, Astacus leptodactylus, in several populations in Turkey. *Crustaceana*, **82,** 531-542 (2009).
40	Kozák, P., Buřič, M., Policar, T., Hamáčková, J., & Lepičová, A. The effect of inter-and intra-specific competition on survival and growth rate of native juvenile noble crayfish Astacus astacus and alien spiny-cheek crayfish Orconectes limosus. *Hydrobiologia*, **590,** 85-94 (2007).

41	Phillips, I. D., Vinebrooke, R. D., & Turner, M. A. Ecosystem consequences of potential range expansions of Orconectes virilis and Orconectes rusticus crayfish in Canada—a review. *Environ. Rev.,* **17,** 235-248 (2009).
42	Freeman, M. A., Turnbull, J. F., Yeomans, W. E., & Bean, C. W. Prospects for management strategies of invasive crayfish populations with an emphasis on biological control. *Aquat. Conserv. Mar. Freshw. Ecosys.*, **20,** 211-223 (2010).
43	Barbaresi, S., & Gherardi, F. The invasion of the alien crayfish Procambarus clarkii in Europe, with particular reference to Italy. *Biol. Invasions*, **2,** 259-264 (2000).
44	Gherardi, F., & Daniels, W. H. Agonism and shelter competition between invasive and indigenous crayfish species. *Can. J. Zool.*, **82,** 1923-1932 (2004).
45	Cruz, M. J., & Rebelo, R. Colonization of freshwater habitats by an introduced crayfish, Procambarus clarkii, in Southwest Iberian Peninsula. *Hydrobiologia*, **575,** 191-201 (2007).
46	Jaklič, M., & Vrezec, A. The first tropical alien crayfish species in European waters: the redclaw Cherax quadricarinatus (Von Martens, 1868)(Decapoda, Parastacidae). *Crustaceana*, **84,** 651-665 (2011).
47	McCormack, R. B. *The Freshwater Crayfish of New South Wales Australia.* (Australian Aquatic Biological 2008).
48	Weinländer, M., & Füreder, L. Associations between stream habitat characteristics and native and alien crayfish occurrence. *Hydrobiologia*, **693,** 237-249 (2012).
49	Holdich, D., & Black, J. The spiny-cheek crayfish, Orconectes limosus (Rafinesque, 1817)[Crustacea: Decapoda: Cambaridae], digs into the UK. *Aquat. Invasions*, **2,** 1-15 (2007).
50	McClain, W. R., & Romaire, R. P. *Procambarid crawfish: life history and biology.* (Southern Regional Aquaculture Center 2007).
51	Penn, G. H. The genus Cambarellus in Louisiana (Decapoda, Astacidae). *Am. Midl. Nat.*, **44,** 421-426 (1950).
52	Abrahamsson, S. A. Density, growth and reproduction in populations of Astacus astacus and Pacifastacus leniusculus in an isolated pond. *Oikos*, **22,** 373-380 (1971).
53	Hendrix, A. N., & Loftus, W. F. Distribution and relative abundance of the crayfishes Procambarus alleni (Faxon) and P. fallax (Hagen) in southern Florida. *Wetlands*, **20,** 194-199 (2000).
54	Acosta, C. A., & Perry, S. A. Spatio-temporal variation in crayfish production in disturbed marl prairie marshes of the Florida Everglades. *J. Freshw. Ecol.*, **17,** 641-650 (2002).
55	Jones, C. M., McPhee, C. P., & Ruscoe, I. M. A review of genetic improvement in growth rate in redclaw crayfish Cherax quadricarinatus (von Martens) (Decapoda: Parastacidae). *Aquac. Res.*, **31,** 61-67 (2000).
56	Patoka, J., Wardiatno, Y., Kuříková, P., Petrtýl, M., & Kalous, L. Cherax quadricarinatus (von Martens) has invaded Indonesian territory west of the Wallace Line: evidences from Java. *Knowl. Manag. Aquat. Ecosyst.*, **417,** 39 https://doi.org/10.1051/kmae/2016026 (2016).
57	Wygoda, M. L. Notes on terrestrial activity of the crayfish Procambarus alleni (Faxon) in west-central Florida. *Fla. Sci.*, **44,** 56-59 (1981).
58	Souty-Grosset, C., Holdich, D. M., Noël, P. Y., Reynolds, J. D., & Haffner, P. *Atlas of Crayfish in Europe*. (Museum national d'Histoire naturelle, Paris 2006).
59	Mills, B. J., & Geddes, M. C. Salinty tolerance and osmoregulation of the Australian freshwater crayfish Cherax destructor Clark (Decapoda: Parastacidae). *Mar. Freshw. Res.*, **31,** 667-676 (1980).
60	Black, J. B. Cyclic male reproductive activities in the dwarf crawfishes Cambarellus shufeldti (Faxon) and Cambarellus puer Hobbs. Trans. *Am. Microsc. Soc.*, **85,** 214-232 (1966).
61	Nunes, A. L., Zengeya, T. A., Hoffman, A. C., Measey, G. J., & Weyl, O. L. Distribution and establishment of the alien Australian redclaw crayfish, Cherax

	quadricarinatus, in South Africa and Swaziland. *PeerJ*, **5,** e3135 https://doi.org/10.7717/peerj.3135 (2017).
62	Mosig, J. *The Australian Yabby Farmer.* (Landlinks Press 1998).
63	Veselý, L. *et al.* Salinity tolerance of marbled crayfish Procambarus fallax f. virginalis. *Knowl. Manag. Aquat. Ecosyst.*, **418,** 21 (2017).
64	Kang, S. R., & King, S. L. Influence of salinity and prey presence on the survival of aquatic macroinvertebrates of a freshwater marsh. *Aquat. Ecol.*, **46,** 411-420 (2012).
65	Coughran, J., & Daly, G. Potential threats posed by a translocated crayfish: the case of Cherax destructor in coastal drainages of New South Wales, Australia. *Crustacean Res.*, **7,** 5-13 (2012).
-	Only species possessing this trait are mentioned in literature, without references species are not expected to possess such traits
**Expert judgement**	
a	Similar to C. puer: 0-5
b	Only lives in single inland freshwater lake area, no salinity tolerance expected
c	Similar to C. puer: 2
d	Similar to other Cherax: 2
e	Similar to C. puer: Low
f	Similar to C. papuanus: Low
g	Similar to C. patzcuarensis: Low
h	Reports of slow growth from aquarium pet owners: https://tropischevissengids.jouwweb.nl/lagere-diersoorten/kreeften/blue-moon (slow), https://sites.google.com/site/rivierkreeften/aquariumhobby (slow), https://aquaticarts.com/products/supernova-crayfish (slow), https://tropischevissengids.jouwweb.nl/lagere-diersoorten/kreeften/zebrakreeft (slow)
i	Mean of other Procambarus: medium
j	Similar to C. diminitus: restricted range
k	Only lives in single inland lake area, low migration
l	Similar to other Procambarus: yes
m	Similar to C. quadricarinatus: 4-5
n	Similar to C. puer: low aggression
o	Reports of aggression from aquarium pet owners: https://tropischevissengids.jouwweb.nl/lagere-diersoorten/kreeften/blue-moon (medium) https://aquaticarts.com/products/supernova-crayfish(medium), https://sites.google.com/site/rivierkreeften/aquariumhobby (low)
p	Reportedly not aggressive from aquarium pet owners: https://tropischevissengids.jouwweb.nl/lagere-diersoorten/kreeften/zebrakreeft (low/medium)
q	Similar to C. patzcuarensis: vector with low susceptibility
r	Similar to other Cherax: high susceptibility

Supplementary Table S3. Literature references and expert judgement descriptions for Supplementary Table S2
